# A Designed Conformational Shift To Control Protein Binding Specificity**

**DOI:** 10.1002/anie.201403102

**Published:** 2014-08-12

**Authors:** Servaas Michielssens, Jan Henning Peters, David Ban, Supriya Pratihar, Daniel Seeliger, Monika Sharma, Karin Giller, Thomas Michael Sabo, Stefan Becker, Donghan Lee, Christian Griesinger, Bert L de Groot

**Affiliations:** Computational Biomolecular Dynamics Group, Max Planck Institute for Biophysical ChemistryAm Faßberg 11, 37077 Göttingen (Germany); NMR-based Structural Biology, Max Planck Institute for Biophysical ChemistryGöttingen (Germany)

**Keywords:** molecular dynamics, protein design, protein–protein interactions, ubiquitin

## Abstract

In a conformational selection scenario, manipulating the populations of binding-competent states should be expected to affect protein binding. We demonstrate how in silico designed point mutations within the core of ubiquitin, remote from the binding interface, change the binding specificity by shifting the conformational equilibrium of the ground-state ensemble between open and closed substates that have a similar population in the wild-type protein. Binding affinities determined by NMR titration experiments agree with the predictions, thereby showing that, indeed, a shift in the conformational equilibrium enables us to alter ubiquitin’s binding specificity and hence its function. Thus, we present a novel route towards designing specific binding by a conformational shift through exploiting the fact that conformational selection depends on the concentration of binding-competent substates.

Conformational plasticity plays a crucial role in protein function, often determining the rate of enzyme catalysis[1–5] as well as protein–protein[6] and protein–ligand recognition.[7] A well-known example in enzyme catalysis is dihydrofolate reductase,[1] where every intermediate state in the enzymatic cycle possesses lowly populated states that are connected to the adjacent states within the catalytic scheme. Several other enzymes are known[2–4] where the turnover rate is sensitive to modifications in the enzyme’s conformational distribution. As for molecular recognition, fluctuations within the ground-state ensemble of the protein, which are compatible and incompatible with binding, can influence the binding affinity. For example, an interleukin-2 (IL-2) mutant with a shift towards a more binding compatible conformational substate in the unbound ensemble was demonstrated to have increased binding affinity for its binding partner IL-2Rβ.[6] The naturally arising question is how conformational equilibria can be manipulated to alter or adjust protein functionality. In this work, we show how the rational modulation of different substate populations in the ground-state ensemble of ubiquitin can be used to achieve selective binding.

Ubiquitin is an important signaling molecule involved in a myriad of signaling pathways through the binding of a diverse set of receptors. More than 150 cellular proteins are estimated to interact noncovalently with ubiquitin.[8] Structurally, ubiquitin consists of one five-stranded β-sheet and one short (1.5 turn) and one long (3.5 turn) α-helix.[9,10] Previous NMR and molecular dynamics studies revealed how such a small and structurally simple protein recognizes a diverse set of receptors: the global conformational ensemble of unbound ubiquitin covers the same conformational space found in ubiquitin complexes, suggesting conformational selection as the primary recognition mechanism.[11] Moreover, a single dominant collective motion in ubiquitin was revealed that covers the motion observed in unbound ubiquitin as well as the motion observed in the ensemble of ubiquitin complexes (X-ray structures). This motion, termed the pincer mode, describes the transition between an open and a closed ubiquitin substate. For nine out of eleven ubiquitin complexes studied previously,[12] partners interact preferentially with either the open or the closed substate.

The conformational preference of binding partners for either the open or the closed ubiquitin substate opens the possibility for a novel computational design strategy: rather than optimizing the binding interface, the conformational preference of ubiquitin is shifted to achieve selective binding (Figure 1 A). In native ubiquitin, both substates are similarly populated, allowing complex formation with binding partners that require either the open or the closed substate. Modifying the dynamics such that only one substate is populated should result in selective binding. Our computational protocol (Figure 1 B) serves to design point mutants introducing a conformational shift in the ground-state ensemble. Previous attempts through a combination of computational design and phage display library screening identified potential mutations to achieve a similar effect in ubiquitin.[13,14] However, in these cases at least six combined mutations were required and the mutations were selected based on their affinity for binding partners, not based on the conformational shift in the ground-state ensemble as done here. The results in those previous studies are difficult to interpret in terms of conformational equilibria. In one case, the resulting mutations mainly change the kinetics, which were analyzed using only simple kinetic models,[14] and not the conformational equilibrium. In another case, the mutations combined for a reduction in conformational entropy (by introduction of disulfide bonds), conformational stabilization, and surface mutations,[13] which make it impossible to disentangle the effects. In other recent work[15] several mutants to modulate the ubiquitin system have been identified based on binding affinity. In this functional study the details of the molecular mechanism were secondary and therefore not investigated in detail. In the present study we aim at inducing a conformational shift by selecting mutants solely according to their population along the pincer mode.

**Figure 1:**
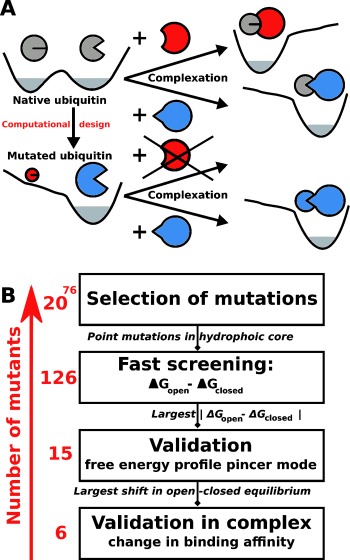
A) Free native ubiquitin has two dominant substates: open and closed. Binding to different binding partners can occur in either the open or the closed substate depending on the binding partner. Ubiquitin is mutated by computational design to stabilize one of the two substates. These mutants are expected to bind selectively to only one class of binding partners. The gray area in the free energy surface indicates the ground state population. B) Computational protocol used to identify mutations shifting the conformational equilibrium and their effect on binding. Fast screening and validation of ubiquitin in the complexes is done using a free energy computational protocol based on the Crooks–Gaussian intersection method. Umbrella sampling simulations were used to compute the free energy profile along the pincer mode. Color code: Blue: stabilized in the open substate or complex binding protein in open substate; red: stabilized in the closed substate or complex binding protein in closed substate; gray: no preference. This color code is maintained in all figures, including the Supporting Information.

An automated, thermodynamic free energy based computational screening approach[18] is proposed to identify point mutations stabilizing ubiquitin in either of the two substates (Figure 1 B, for computational details see the Supporting Information). Ubiquitin consists of 76 amino acids, resulting in 19×76=1444 potential point mutations. From these, we selected mutations according to two criteria. First, only mutations in the hydrophobic core were selected (14 positions; Table S1 in the Supporting Information), such that the atomic interactions at the binding interface are left unperturbed. Second, we decided to insert only hydrophobic residues, as well as serine and threonine, because the insertion of charged or more polar residues would potentially destabilize ubiquitin. Also glycine and tryptophan were excluded because the size of these amino acids could substantially distort the hydrophobic core of ubiquitin. Applying these selection criteria, we obtained 126 mutants that were computationally tested for changes in their open–closed equilibrium compared with native ubiquitin (Figure 2). Screening of 126 mutations still requires substantial computing time. In Section S1.7 in the Supporting Information we describe an approach to reduce the number of candidate mutations using functional mode analysis;[16,17] this approach successfully identified the positions of the most promising mutations based on ten 100 ns molecular dynamics (MD) simulations of unbound ubiquitin.

**Figure 2:**
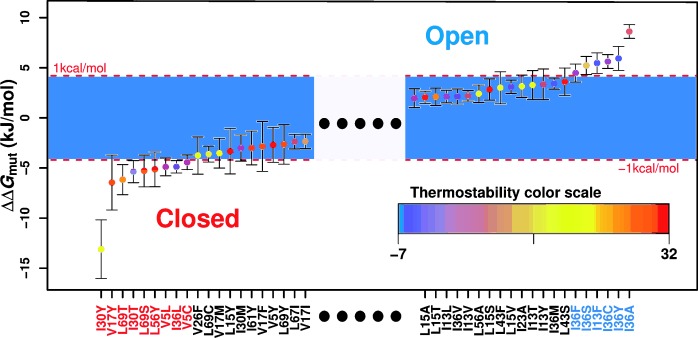
Conformational preference of ubiquitin mutants calculated using FGTI/CGI. For clarity, only the 20 mutants demonstrating the largest stabilization of either the open or closed substate are shown (a full overview of all 126 mutations including their thermal stability can be found in Figure S5). The inset gives the color coding for the folding free energy. The error bars represent the uncertainty of the values estimated using bootstrapping.

Of the 126 mutations examined, 15 cases resulted in a significant (>4.184 kJ mol^−1^ or 1 kcal mol^−1^) relative stabilization of either the open or closed substate (Figure 2). In addition, the change in folding free energy was estimated[18] to monitor potential destabilization of the protein. As expected, most mutants mildly destabilize wild-type ubiquitin. For the 15 most promising mutations, the destabilization is less than 23.6 kJ mol^−1^, the folding free energy of the wild-type ubiquitin.[19] An additional computational validation was performed using umbrella sampling simulations (for computational details see the Supporting Information). Although this method requires orders of magnitude more computational time, the methodology provides a complete free energy profile along the pincer mode (see Figure 3, and Figures S8 and S9 in the Supporting Information). Overall, 11 out of the 15 mutations were confirmed by umbrella sampling simulations, and of those 15 mutants I13F, I36F, I36Y,and I36A show significant stabilization of the open substate, whereas L69S and L69T show significant stabilization of the closed substate. A recent study[20] found all six of these mutants to have a negative effect on yeast growth rate, indicating that they significantly interfere with normal ubiquitin function. Also, the L69S mutant was previously described by Fushman and co-workers[19] as a more selective binder than wild-type ubiquitin. We were able to identify the selectivity of this mutant in terms of a shift in the open–closed equilibrium.

**Figure 3:**
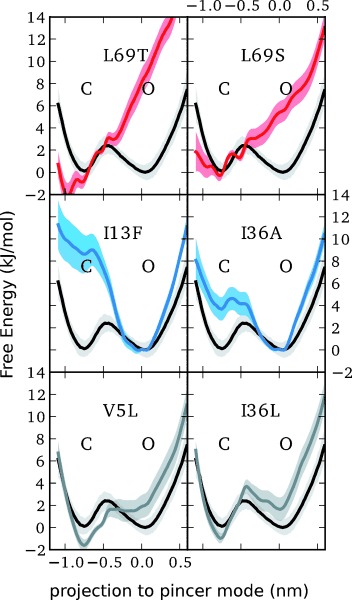
Free energy profiles for six different ubiquitin mutants, calculated using umbrella sampling simulations. Mutants preferring the closed substate are shown in red, open substate stabilizing mutants are depicted in blue, those without a preference are shown in gray. The wild-type profile is plotted in black. C refers to the closed substate, O to the open substate.

To test the hypothesis that the identified mutants indeed affect binding selectivity by favoring a specific substate along the pincer mode, the change in binding free energy was calculated for six complexes. Three complexes with ubiquitin predominantly in the open substate (PDB: 1xd3, 2fif, and 4un2), two with ubiquitin predominantly in the closed substate (PDB: 1nbf and 2g45), and one complex without preference for either the open or closed substate (PDB: 2hth) were chosen for this assessment. The accuracy of the procedure was ensured by using closed thermodynamic cycles, as well as by comparing to known experimental data (see Sections S2.1–S2.3 in the Supporting Information). Seven ubiquitin mutations were performed on each of these complexes, three preferring the open substate (I13F, I36A, I36Y), two preferring the closed substate (L69S, L69T), and two that populate the complete ground-state ensemble similar to the wild-type (V5L, I36L). The V5L and I36L mutants, although initially identified as stabilizing the closed substate, were revealed by umbrella sampling simulations to have almost no effect on the substate populations (Figure S8).

Assuming that the pincer mode is indeed the factor determining binding specificity, we would expect that the binding affinity would decrease if the preferred substate of ubiquitin in the complex and the conformational preference of the mutant were not compatible. Stabilizing ubiquitin in the substate compatible with binding would result in a marginal increase in affinity because in wild-type ubiquitin each of the substates has a substantial population. The only gain that can be expected in binding affinity is by alleviating the entropic cost of depleting the noncompatible substate. Assuming a similar population of both substates, this cannot exceed −1.7 kJ mol^−1^ (=*RT* ln(*p*_wt_/*p*_mut_), with *p*_wt_ the population of the binding compatible substates in the wild-type (≈0.5) and *p*_mut_ the population of these substates in the mutant). Destabilization by depopulation of the binding-compatible conformation, on the other hand, can fully abrogate binding, and is only limited by the preference of this substate in the complex or the binding free energy. Strikingly, as can be seen in Figure 4, most of the mutations that prefer a pincer mode conformation that is incompatible with the complex formation indeed destabilize the complex substantially. The average change in binding free energy is ΔΔ*G*_binding,compatible_=2.3±4.0 kJ mol^−1^ for the mutants with a conformational preference that is compatible with the complex, ΔΔ*G*_binding,neutral_=1.2±2.9 kJ mol^−1^ for complexes or mutants without conformational preference, and ΔΔ*G*_binding,incompatible_=9.0±5.2 kJ mol^−1^ for mutants that are incompatible. The difference between the neutral versus incompatible complexes and compatible versus incompatible complex is statistically significant within a 99 % confidence level according to a t-test performed on the data. The predicted changes in binding free energy correlate well with the free energy shift calculated from umbrella profiles (Figure S13) even though this comparison does not take into account any effects of the mutation except that on the population along the pincer mode. The only extreme outlier in the group predicted to have lower binding affinity (the open mutant I36A in the closed complex 1nbf) involved the mutation which showed the weakest population shift (Table S4). Apart from the results predicted in this study, this model can also explain several cases from available literature on ubiquitin mutants with a modified binding profile (Figure S12).

**Figure 4:**
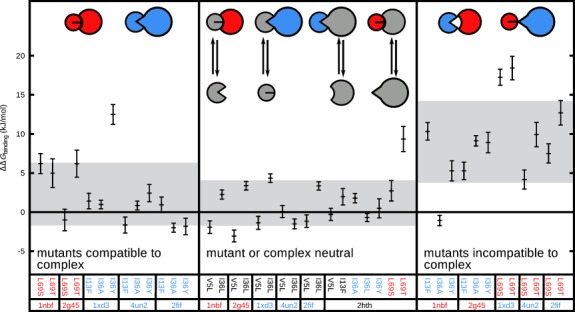
Prediction of binding free energy differences between wild-type ubiquitin and different point mutations (ΔΔ*G*_binding_=Δ*G*_binding,mutant_−Δ*G*_binding,wild-type_) calculated using FGTI/CGI. Positive ΔΔ*G*_binding_ indicates a decrease in binding affinity. Combinations of mutants and binding partners have been divided into three groups. In cases where the mutant stabilizes the state that is compatible to binding (left-most category), a slight increase in binding affinity is expected, but this seems to be too weak to be detected by simulation. The middle section of the graph contains combinations where at least the mutant and/or the binding partner do not prefer one state of ubiquitin. Here, no change in binding free energy is observed, as expected. The right-most section of the graph contains combinations where the population shift caused by the mutation is expected to decrease binding affinity. This is indeed the case for most of the combinations. The gray area gives an indication of the distribution of the data, the middle is the mean, the width is twice the standard deviation. The error bars represent the uncertainty of the values estimated using bootstrapping.

To validate the predicted effects, we performed NMR-based titrations using ^15^N isotopically labeled wild-type and mutant ubiquitin with unlabeled dsk2 (the binding partner in structure 4un2) as the interaction partner. NMR-based titrations are well suited to determine affinities for protein–protein interactions. Here, ^15^N chemical shifts for ubiquitin and ubiquitin mutants were monitored as a function of increasing dsk2 concentration. These chemical shift perturbations were then used to determine global dissociation constants (see the Supporting Information). When we compared the change in affinity between the studied ubiquitin mutants and wild-type ubiquitin with dsk2, an agreement (Figure 5) was found, demonstrating the direct validation of the novel computational method employed here by solution experiments.

**Figure 5:**
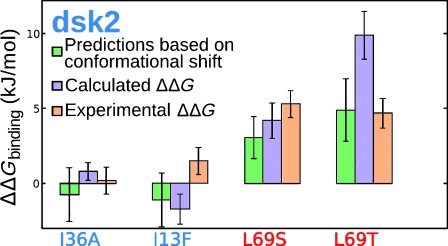
Comparison of change in binding free energy predicted from the conformational shift in unbound ubiquitin (see Section S1.8) with the calculated results for ubiquitin (using FGTI/CGI) in complexes and the experimental result. For the prediction, the population in both states was estimated from the free energy profiles calculated by umbrella sampling (Figure 3).

The modulation of protein–protein binding by means of a conformational shift offers some exciting opportunities for protein design. In systems like ubiquitin that interact with different binding partners in different conformations, our approach can be used to introduce selectivity, as shown here. In extension, for wild-type proteins binding in weakly populated conformations, the same approach could be used to significantly raise the binding affinity by increasing the binding-compatible population.

In this work, a proof of principle is presented that selective protein–protein binding can be achieved by modifying conformational equilibria rather than optimizing the binding interface. Using ubiquitin as a case study, the computational protocol was complemented by experimental validation and was shown to yield selective binding by means of a conformational shift due to designed single-residue mutations. Just as conformational plasticity emerged during evolution to insert and adapt functionality in proteins, conformational plasticity can be controlled by computational design to alter functionality. We note that the present computational approach to identify critical mutations remote from the interface may present a first step towards a designed allosteric switch.[21]
