# Supplementary material for: A Designed Conformational Shift To Control Protein Binding Specificity
Source: Angew Chem Int Ed Engl. 2014 Aug 12;53(39):10367–71. doi: 10.1002/anie.201403102 (PMC4497613; doi:10.1002/anie.201403102)
Supplement: Supplementary file 1 [file anie0053-10367-sd1.pdf]

Angewandte Chemie
Eine Zeitschrift der Gesellschaft Deutscher Chemiker

Supporting Information

© Wiley-VCH 2014

69451 Weinheim, Germany

A Designed Conformational Shift To Control Protein Binding Specificity**

*Servaas Michielssens, Jan Henning Peters, David Ban, Supriya Pratihar, Daniel Seeliger, Monika Sharma, Karin Giller, Thomas Michael Sabo, Stefan Becker, Donghan Lee, Christian Griesinger, and Bert L. de Groot**

anie_201403102_sm_miscellaneous_information.pdf

S1 Methods

S1.1 Definition of the pincer mode

The pincer mode is described as the first eigenvector of a PCA based on the 213 backbone atoms of residues 1-71 of 61 experimentally determined ubiquitin structures taken from the Protein Database (PDB: 1aar, 1cmx, 1f9j, 1nbf, 1ogw, 1p3q, 1s1q, 1sif, 1tbe, 1ubi, 1ubq, 1uzx, 1wr6, 1wrd, 1xd3, 1yd8, 2ayo, 2c7m, 2c7n, 2d3g, 2dx5, 2fid, 2g45, 2gmi, 2hd5, 2hth, 2ibi, 2j7q, 2jf5, 2o6vm, 2ojr, 2oob, 2qho, 2wdt, 2wwz, 2wx0, 2wx1m, 2zcb, 2zcc, 2znvc, 2znv, 2zvn, 2zvo, 3a1q, 3a33, 3a9jb, 3a9kb, 3by4, 3c0r, 3dvgx, 3dvnxm, 3efu, 3h7pb, 3h7s, 3hm3, l3ihp, 3jsvb, 3jvz, 3jw0, 3ldz, 3mhs, 3mtn).

S1.2 Simulation parameters

All simulations were carried out with GROMACS,[1] the AMBER99sb force field,[2] the SPC-E water model[3] and ion parameters from Joung *et al.*[4] The simulation system consisted of ubiquitin solvated in a dodecahedron box with 150 mM NaCl. For the equilibrium simulation a leap-frog integrator was used with the thermostat of Bussi *et al.*[5] to keep the temperature at 300 K and a coupling constant of 0.1ps. A constant pressure of 1 atm was achieved using the Berendsen barostat[6] with a coupling constant of 1ps. Electrostatic interactions were calculated at every step with the particle-mesh Ewald method.[7] Short-range repulsive and attractive dispersion interactions were simultaneously described by a Lennard-Jones potential with a cutoff of 1.1 nm and a switching function between 1.0 and 1.1 nm. Dispersion corrections for energy and pressure were applied. The SETTLE[8] algorithm was used to constrain bonds and angles of water molecules, and LINCS[9] was used for all other bonds. Virtual sites[10] were introduced to remove other fast vibrating degrees of freedom, allowing a time step of 4 fs.

S1.3 Screening of ubiquitin mutants using FGTI/CGI

The initial screening of the 126 point mutations was based on a computational protocol using fast growth thermodynamic integration (FGTI) with the Crooks Gaussian inter-

section method (CGI).[11] The aim of this screening was to compute $\Delta\Delta G$, which is the shift in stability of the open versus the closed substate relative to wild type ubiquitin. This requires a definition of the open and closed substate. As can be observed in figure S2, ubiquitin possesses two minima along the pincer mode, one corresponding to an open substate (right), the other to a closed substate (left). Between these two substates there is a barrier of approximately 2 kJ/mol (figure S2). As this is less than the thermal energy at 300 K which is (approximated for an ideal gas) $3/2 \cdot N_A \cdot kT \approx 3.74$ kJ/mol, a simulation of the protein at room temperature will frequently cross this barrier. This is undesirable in the CGI simulations, as we try to analyze the effect of the mutation in the open and closed substates individually. To restrict the protein to either the open or the closed substate, a modified version of the Essential Dynamics code of GROMACS[12–14] was used to add semi-harmonic potentials (equation 1 or a "mirrored" version) along the collective pincer mode coordinate ξ (figure S2)).

$$V(\xi) = \begin{cases} V_0 \cdot (\xi - \xi_0)^2 & \xi > \xi_0 \\ 0 & \xi \leq \xi_0 \end{cases} \tag{1}$$

This potential is necessary to prevent the simulation from leaving its assigned substate, whereas the influence of the potential on the simulation should otherwise be as small as possible. To achieve this, a high force constant $V_0 = 1000\ kJ/(mol \cdot nm^2)$ was selected. The resulting high force acting on the system, if it enters the non-zero part of the potential, will reduce the time the simulation spends in this region. The position of the external potentials have been chosen to be offset from the center of the energy barrier observed in unbound wild-type ubiquitin. This was done to prevent the creation of an artificial energy minimum in cases where the mutation shifts the position of this barrier. 14 hydrophobic core residues (Table S4) of ubiquitin were chosen and mutated to the hydrophobic residues Alanine (A), Valine (V), Isoleucine (I), Leucine (L), Methionine (M), Phenylalanine (F), Tyrosine (Y) and Cysteine (C) as well as Serine (S) and Threonine (T), resulting in 126 mutants.

The thermodynamic cycle in figure S3 is used to determine the free energy difference

($\Delta G_{closed} - \Delta G_{open}$) caused by a mutation. Fast growth thermodynamic integration (FGTI) evaluated using the Crooks Gaussian intersection (CGI) was used to determine ΔG_3 and ΔG_4. This gives us access to $\Delta G_2 - \Delta G_1$ wich is equal to $\Delta G_{closed} - \Delta G_{open}$. From the simulation ensemble of unbound ubiquitin (based on the X-ray structure 1UBI) ten open and ten closed structures were selected and mutated using the software WHATIF.[15] Open structures were selected equally distributed along the pincer mode from 1 nm to -0.2 nm and closed from -1 nm to -0.65 nm. Each of these mutated structures was simulated for 10 ns at equilibrium conditions (resulting in a total simulation time of 200 ns for each mutant, and 25.2 μs for all mutants), the ten open structures with the external potential restricting them to the open substate and ten closed ones with the potential restricting them to the closed substate. From these equilibrium ensembles, 100 snapshots were extracted from each of the open and the closed ensembles for 100 ps FGTI simulations using tools described in the previous work.[16] To compensate for the fact that most of the mutations studied here belong to a class that has been found to be computationally challenging, we significantly increased sampling (from 50 ps to 100 ps per transition). The integrated $\partial H/\partial\lambda$ values were then used to determine the free energy difference using the CGI method.[11]

FGTI/CGI can be also be used to calculate the change in thermodynamic stability of a mutant,[16] that is the change in the folding free energy of the protein due to the mutation. For this, the free energy change of a mutation calculated in the folded protein is compared to that of the unfolded protein, which can be estimated from the free energy change of a GXG (Glycine-X-Glycine) tripeptide.[16]

S1.4 Umbrella sampling simulations

Validation of the most promising mutations was done using umbrella sampling simulations along the pincer mode. Along this collective coordinate, 21 equally spaced umbrella windows were placed. Those windows were placed ranging from -1 to 0.5 nm. The initial structure in each window was selected as the structure closest to the umbrella minimum from a free MD simulation of ubiquitin. Again WHATIF[15] was used to introduce the

mutations. A force constant of 1000 kJ/(mol nm^2) was applied, using the pincer mode as a reaction coordinate. In each of the windows, 11 times 15 ns of simulation were performed, starting each time with different random velocities. Only the last 10 ns were used for analysis. The total simulation time for umbrella sampling simulations was 3.5 μs. The free energy profile was determined using WHAM[17] and the error was estimated using Bayesian bootstrapping of complete histograms as implemented in g_wham.[18]

S1.5 Calculating binding free energy changes due to mutation of ubiquitin

For each complex, five structures equally distributed along the pincer mode were selected from the equilibrium simulation (performed with wild type ubiquitin). Each of these structures were mutated using the software WHATIF[15] and simulated for 110 ns using the GROMACS 4.5 simulation package.[1] The first 10 ns are not used in subsequent steps to allow the protein structure to relax into the ground state of the mutated ubiquitin. Of the remaining 100 ns per trajectory, 50 snapshots (every 2 ns) containing coordinates of all atoms of the simulation system (including solvent water and ions) were extracted and used to set up Fast Growth Thermal Integration (FGTI) simulations in which the mutated residue was changed back to the wild type residue according to a setup adapted from previous studies.[16] Similarly, 300 structures were selected from the equilibrium simulation of wild type ubiquitin in the complex and FGTI simulations were performed in which the wild type protein was turned into the mutant.

For two reasons, the equilibrium simulations of ubiquitin in complexes are chosen to be significantly longer (5x100 ns instead of 10x10 ns) than simulations in free ubiquitin. First, the simulation system including the binding partner is larger than that only consisting of ubiquitin, hence it can be assumed that more time is needed to sample the conformational space. Secondly, the goals and hence requirements of the two calculations are different. The calculations on unbound ubiquitin were a screening process that was supposed to identify valid candidates from a set of 126 candidates. Promising mutations were then validated using umbrella sampling. The calculations here are performed on a

smaller set of systems (six mutations in four complexes resulting in 24 combinations) to validate the influence on the binding behavior. A longer equilibration, expected to lead to more accurate energy estimates, was also chosen because a further validation using umbrella sampling, as carried out for isolated ubiquitin, was not feasible for ubiquitin complexes.

S1.6 Protein Sample Preparation and NMR measurements

Uniformly ^{15}N-labelled ubiquitin was expressed recombinantly in *Escherichia Coli* and was purified as described before.[19] Ubiquitin mutants were generated by PCR-based site-directed mutagenesis using the QuikChange II-Kit (Agilent) following the instructions of the supplier. The ubiquitin-associated domain (UBA) of the yeast protein dsk2 (residues 328-373) was cloned into a modified pET28a (Novagen) expression vector containing a His_7-tag and a TEV cleavage site upstream of the multiple cloning site. Expression was performed in *Escherichia Coli* strain Bl21(DE3). The protein was purified by immobilized metal affinity chromatography on Ni-NTA resin (Qiagen) followed by TEV cleavage to remove the His7-tag, and gel filtration. For NMR titration experiments both ubiquitin and the UBA domain of dsk2 were dialyzed against the same buffer (50 mM sodium phosphate, pH 6.5, 100 mM sodium chloride and 0.1 % (w/w) sodium azide). The concentration of ubiquitin and Dsk2 UBA was adjusted to 1 mM and 2 mM, respectively. NMR titrations were performed by monitoring the chemical shifts (ω) of ^{15}N-labeled wild type and ubiquitin mutants (I13F, I36A, L69S, L69T) using [^{15}N, ^{1}H]-HSQCs collected at increasing concentrations of unlabeled dsk2. For all titrations the concentration of ^{15}N-labeled ubiquitin was kept constant at 100 μM. As the concentration of unlabeled dsk2 is increased, the monitored perturbations (figure S10) in the ^{15}N chemical shift for a given ubiquitin mutant's backbone resonance permits the extraction of a dissociation constant (K_D) and the ω value at saturation assuming a two-state binding model.[20] For a given ubiquitin mutant, all quantifiable resonances were globally fit to render one K_D. For the I13F, I36A, L69S, L69T ubiquitin mutants a total of 26, 30, 29, and 28 ^{15}N resonances were used in the global determination of each ubiquitin mutant's K_D, respectively. A

total of 24 resonances were used for the K_D determination of wild type ubiquitin. The global K_Ds for wild type ubiquitin and the I13F, I36A, L69S, and L69T ubiquitin mutants are 12 ± 4 μM, 22 ± 4 μM, 13 ± 3 μM, 99 ± 11 μM, and 79 ± 10 μM, respectively. The error in the extracted global K_D was determined by assuming a 10% deviation in the concentrations of the stock solutions used for the NMR based titrations.[20] Using this range of error in the stock solutions, a Monte Carlo approach was employed in which 1000 sets of titration curves were randomly generated (each set creates a shift in the abscissa) for all resonances used within a given ubiquitin mutant. All sets of titration curves were then subsequently refit in a global manner. The error in the K_D was taken as the standard deviation from the globally determined K_Ds. [^{15}N, ^{1}H]-HSQCs for the I36A, L69S, and L69T ubiquitin mutants were collected with 128 ($t_{1,max}$ = 53.0 ms) and 1024 ($t_{2,max}$ = 121.7 ms) complex points with 16 transients collected per point. For the I13F ubiquitin mutant, [^{15}N, ^{1}H]-HSQCs were acquired with 150 ($t_{1,max}$ = 48.3 ms) and 1024 ($t_{2,max}$ = 95.0 ms) complex points with 24 transients collected per point. Wild type ubiquitin data was recorded with 100 ($t_{1,max}$ = 32 ms) and 512 $t_{2,max}$ = 48 ms) points in the indirect and direct dimensions, respectively with 16 transients collected per point. All experiments maintained a one second recycle delay and were conducted at 298 K. Titrations for the I36A, L69S, and L69T ubiquitin mutants were performed at a spectrometer operating at a ^{1}H Larmor frequency of 700 MHz and data for the wild type and the I13F mutant was conducted with a 900 MHz spectrometer.

S1.7 Pre-Selecting residues for mutation

In this study, a 14 core residues were screened for the effect of mutations on the pincer mode. With 9 mutations per residue, this results in a total number of 126 mutations, screening of which requires significant computation time. This requirement could be reduced if it was possible to pre-select residues that can be expected to play an important role in pincer mode dynamics.

As an approach to identify such residues, we used function mode analysis (FMA)[21,22] to find the dynamic mode in each residue that best predicts the pincer mode motion.

We divided ten 100ns simulations of unbound ubiquitin (using PDB structure 1UBI as starting structure and simulating as described in section S1.2) into three parts. The first 10ns of each simulation were ignored as relaxation from the xray structure. The next segment of 45ns from each simulation was used for model building, while the last 45ns of each simulation were only used for evaluation. For each residue of the core (see Table S1), the following steps were performed: First, all structures were least-squares fitted to the three main chain atoms of the residue. Then we used Partial-Least-Squares-based FMA (with 10 internal degrees of freedom) to best model the pincer mode dynamics from the coordinates of the residue atoms of the model building part of the trajectories. This model was used to predict the pincer mode projection for the evaluation part of the trajectories. We calculated the Pearson correlation coefficient between this prediction and the actual pincer mode projection (table S6). This correlation coefficient allows a good prediction of the relevance of each residue to pincer mode dynamics.

Indeed, the three core residues with the highest correlation to the pincer mode (L69, I36 and I13) where also those whose mutations (L69S, L69T, I36A, I36F, I36Y and I13F) turned out to have the highest influence on pincer mode dynamics. Hence, a preliminary screening of residues using this approach could drastically decrease the ammount of computation time required to find mutations that control pincer mode dynamics.

S1.8 Estimation on the conformational shift using the umbrella sampling profiles.

The conformational shift was estimated from the umbrella sampling simulations by computing the free energy difference between the open and the closed state, where the border between the open and closed state was taken as the maximum of the barrier between the open and the closed state (i.e. -0.46 nm). The free energy in both states was estimated from the umbrella sampling profiles using the definition of free energy from statistical mechanics. This is $G_S = -RTln(Z)$, with $Z_S = \sum_{i=1}^{N} exp^{-RTG_i}$ the partition function of that state, the sum was terminated when G_i was more than 10kJ/mol larger than the minimal free energy along free energy profile. The error was obtained by error propa-

gation of the umbrella sampling error. As can be seen in figure S6, population shifts calculated by FGTI/CGI and estimated from umbrella profiles correlate well with a correlation coefficient of 0.54($p = 0.0003$). In addition, in all cases where values calculated by FGTI/CGI and umbrella sampling differ significantly are "false positives", i.e. the shift predicted by the screening could not be observed in the more thorough umbrella sampling calculation. This allows the screening to reliably exclude the high number of neutral mutations from further consideration.

S2 Validation of free energy simulations via closed thermodynamic cycles and comparison to experimental data

S2.1 Closed thermodynamic cycles for free ubiquitin simulations

Closed thermodynamic cycles are a common approach to validate methods calculating free energy differences. Closed thermodynamic cycles are simple thermodynamic cycles and make use of the fact that the free energy is a state property. By explicitly calculating each leg of the cycle, a deviation from the expected value of zero for the sum over all legs yields an estimate of the accuracy of the applied method. The simplest closed cycle contains three states (figure S4). Three closed cycles were calculated both with restrictions to the open and the closed substate. With one exception, the free energy difference along a closed path of mutations was close to zero, as expected (table S2), and all values were within 1 kcal (4.184 kJ/mol) from zero.

S2.2 Closed thermodynamic cycles of mutations in ubiquitin complexes

As for unbound ubiquitin, the accuracy of FGTI/CGI results for ubiquitin complexes was tested using closed thermodynamic cycles. Two of the four closed cycles listed in table S3 are near zero within their statistical uncertainty and are all in the range of ±1 kcal/mol (4.184 kJ/mol). This suggests that that chosen protocol provides a similar accuracy as obtained for isolated ubiquitin.

S2.3 Testing calculated changes in binding free energy against experimental values

Wilkinson *et al.*[23] have measured the changes in binding free energy for different ubiquitin mutants to UCH-L3, the protein which is bound to ubiquitin in the 1xd3 structure. Most of the changes of binding free energy described in the paper are small compared to those we aim to find in this study. To test the feasibility of the used protocol to calculate affinity changes due to mutation, calculations of these values were performed. The results are shown in figure S7. Overall, the Pearson correlation coefficient is 0.70 ($p = 0.016$) . The correlation coefficient for charge perserving mutants (i.e. K6R, L8A, K11R, K27R, K33R and I44A) is 0.93 ($p = 0.020$).

L8A shows the greatest difference in binding free energy both in experiment and calculation. This is a promising result, as it corresponds to the level of change we expect to observe in our study. However, it is also obvious that this value significantly increases the correlation observed between the two datasets (leaving out this point reduces the correlation coefficient to 0.33 ($p = 0.18$) and 0.66 ($p = 0.12$) respectively). Note that closed thermodynamic cycles mainly serve as an internal consistency check, assessing if sampling in the simulations is sufficient, which is crucial for demonstrating the validity of the simulation approach to test the principle of protein design via modifying dynamics. To take it one step further and also make predictions that can be validated experimentally, the accuracy of the force field needs to be addressed. This can be done by comparing to

experimental data as shown in Figures 4 and S7.

S3 Additional validation of the FGTI/CGI screening of free ubiquitin

The most promising mutants were validated using umbrella sampling simulations, but as an additional validation another 23 neutral mutants were also checked. For the additional mutants we expect no significant change in the open-closed equilibrium, as can be seen in figure S9.

Although for the neutral and open stabilizing mutations, the FGTI/CGIscreening appeared to be highly successful. For the nine closed stabilizing mutations, only five were confirmed by umbrella sampling simulations. It appears that the setup initially chosen for fast screening was biased towards false positives for stabilizing the closed substate. This bias is caused by the starting structures chosen for the open substate. Structures equally spaced from -0.2 to 1 nm along the pincer mode were selected. This results in starting structures with relative high energy compared to the closed substate starting structures and the MD simulation was too short to fully relax the structures. To verify if this is indeed the reason leading to the false positives, the screening protocol was repeated with structures that where randomly selected from a free MD simulation in open and closed substates. Again ten structures were selected and this time simulated for 15 ns and only the last 10 ns were used for analysis. This protocol was applied for all the mutants initially predicted to stabilize the closed substate, and (table S4, updated $\Delta G_{closed} - \Delta G_{open}$) this resulted in only one false positive (V17Y) mutant and improved the correlation between free energy changes calculated by CGI and from umbrella potentials to 0.70 (p = 0.02), indicating that the starting structures were the main soure of false positives in the initial protocol.

S4 Dynamics of ubiquitin mutants in complexes

The potential of mean force along the pincer mode (figure S11) shows that the conformational equilibrium of mutants in the complex are usually similar to those of the wild type in the same complex. It remains to be determined if this is indicative of the binding partner having a stronger influence on the conformational behavior of ubiquitin than mutation. The equilibrium simulations used to estimate the PMFs in figure S11 were all started from conformations taken from the corresponding bound wild type ensembles, so a bias due to the starting structures cannot be excluded at this time. It should be noted, however, that the starting structures for the unbound mutant simulations were also picked from the unbound wild type ensemble and hence contained a similar starting structure bias that the mutant simulations seem to have overcome quickly.

S5 Additional experimental evidence of selective influence regarding the ubiquitin core mutations on binding behavior

The observation that surface residue mutations can influence the binding behavior of a protein is well established and is often used to identify binding sites (see for example: Wilkinson *et al.*[23]). It has also been observed that mutations of core residues of ubiquitin can selectively change its binding behavior.[24,25] Here, we provide molecular dynamics simulations of those mutants demonstrating that some of them, although not explicitly designed for this purpose, display a shift in the equilibrium distribution along the pincer mode sufficient to achieve selective binding.

Haririnia *et al.*[24] identified mutations (L69S, L67S) that selectively influence the binding behavior even though the mutated residues are not part of the binding interface. Simulations of L69S on both yeast and human ubiquitin show a shift to the closed conformation along the pincer mode (for human ubiquitin see main text Figure 3). The binding partners investigated by Haririnia *et al.*[24] were Rpn10 and S5a (both of which

did not bind the mutated ubiquitin) as well as Rad23 and hHR24A (which did interact with the mutants). In terms of ubiquitin binding motifs, this means that the mutant does not bind to the ubiquitin-interacting motif (UIM) but does still interact with the ubiquitin-associated domain (UBA). This result is consistent with our previous investigation[26] where complexes have been studied including both a UIM (2d3g) and a UBA (2oob) binding motif. It was found that ubiquitin bound to a UIM ubiquitin binding motif (as in the complex 2d3g) strongly prefers open conformations. This mismatch between the L69S mutant mainly occupying the closed substate of the pincer mode and the ubiquitin bound to an UIM motif in the 2d3g ensemble preferring the open substate could explain the inability of this mutant to bind partners containing the same binding motif. However, this does not explain the full change in binding behavior because L67S does not show this conformational shift, but still displays a modified binding profile.

In a more recent study, Zhang *et al.*[25] engineered a number of ubiquitin mutants specifically to increase the binding affinity to ubiquitin-specific protease 7 (USP7, which is the ubiquitin binding partner in structure 1NBF). They found that the mutations that best improved binding affinity to USP7 also significantly decreased binding affinity to several other ubiquitin binding proteins including the ubiquitin carboxyl-terminal esterase L3 (UCHL3), which is the binding partner to ubiquitin in structure 1XD3. The majority of mutants found by Zhang *et al.*[25] contained cysteine residues in positions 7 (sometimes 8) and 69, which formed a disulfide bond. A molecular dynamics simulation based on a crystal structure of one of these mutants (figure S12) shows that the mutation restricts ubiquitin to the closed substate of the pincer mode. This finding is consistent with the results presented here. These findings may explain the ubiquitin mutant's preference for USP7, which requires ubiquitin to be in the closed substate over UCHL3 which requires the open substate (both depicted in figure S12). Another selective ubiquitin mutant found by Zhang *et al.*,[25] lacking the disulfide bond but including mutations of surface residues to optimize binding affinity, does not show the same preference for the closed substate. In this case, optimization of residues at the binding interface is more than likely the explanation for the enhancement in binding affinity as opposed to a shift in

ubiquitin's conformational equilibrium.

In another recent study by the same group,[27] screening for ubiquitin mutations resulting in a higher binding affinity for USP14 (2AYO) again resulted in a restriction of the ubiquitin mutant to the binding compatible conformation (see figure S12). Although a significant increase in binding cannot be expected only from this restriction, it can have a contribution of 1.7 kJ/mol (see main text).

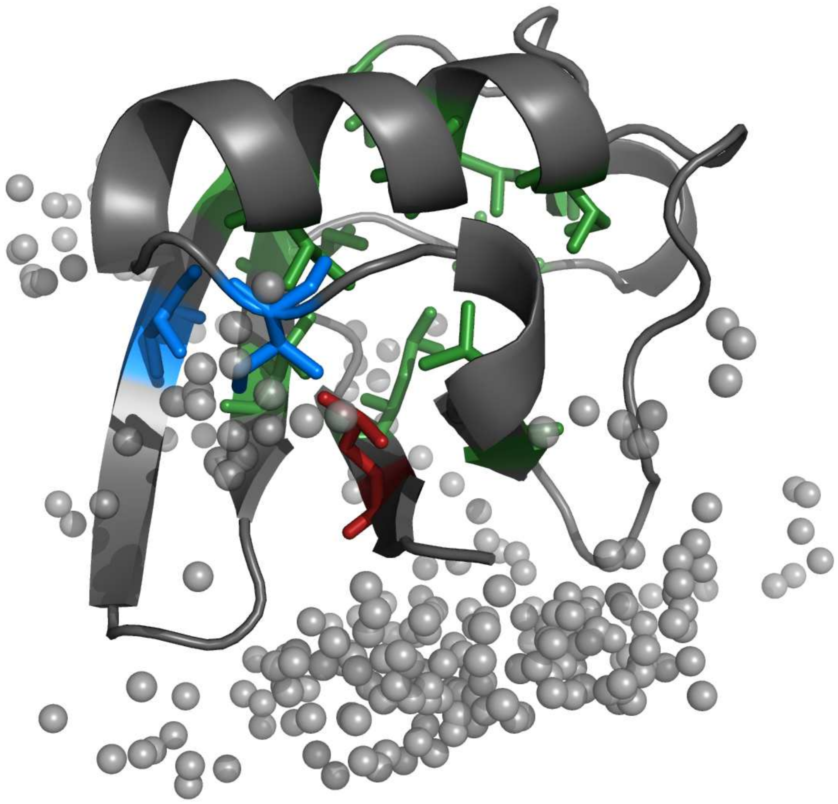

Figure S1: Positions of mutated ubiquitin core residues (in color) - Valine 5 and Isoleucine 36 that have been mutated to stabilize the open substate are colored blue, Leucine 69 that has been mutated to stabilize the closed substate in red. The contacts of the binding partners studied in this paper are shown as grey spheres.

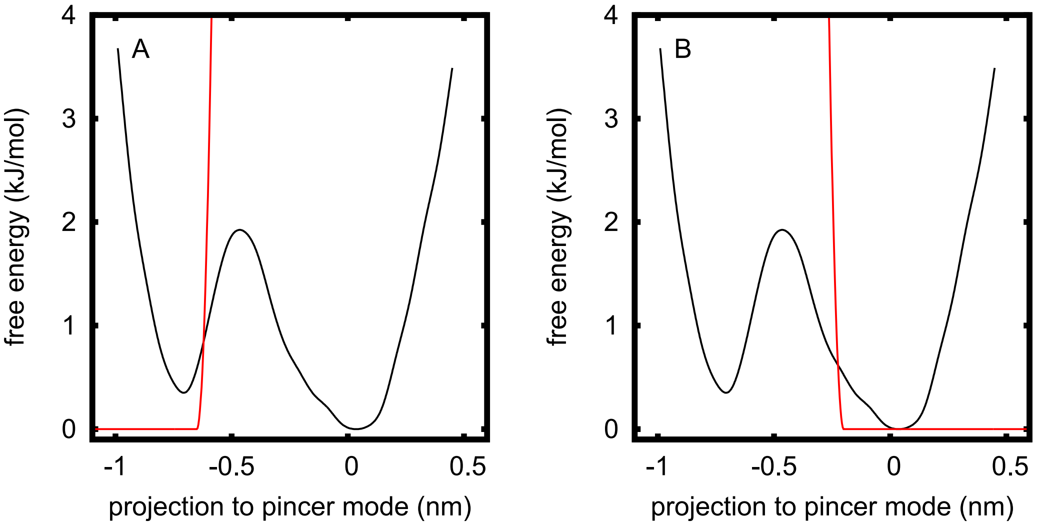

Figure S2: Potentials used to restrict ubiquitin to closed (A) or open (B) conformations. The free energy profile of unbound ubiquitin is plotted in black, the semi-harmonical potential to prevent the simulation to enter the open or closed substate is plotted in red. Due to the high force constant, the non-zero part of the potential is almost vertical.

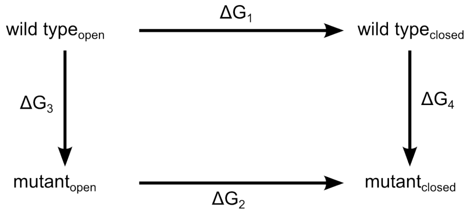

Figure S3: Thermodynamic cycle used to test stabilization of one over the other substate of ubiquitin by a mutation.

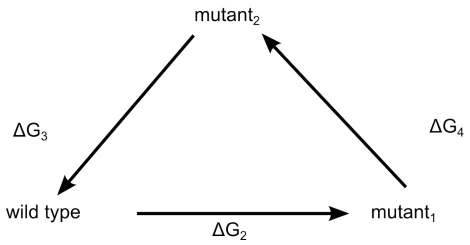

Figure S4: Closed cycle used to validate the free energy calculation protocol.

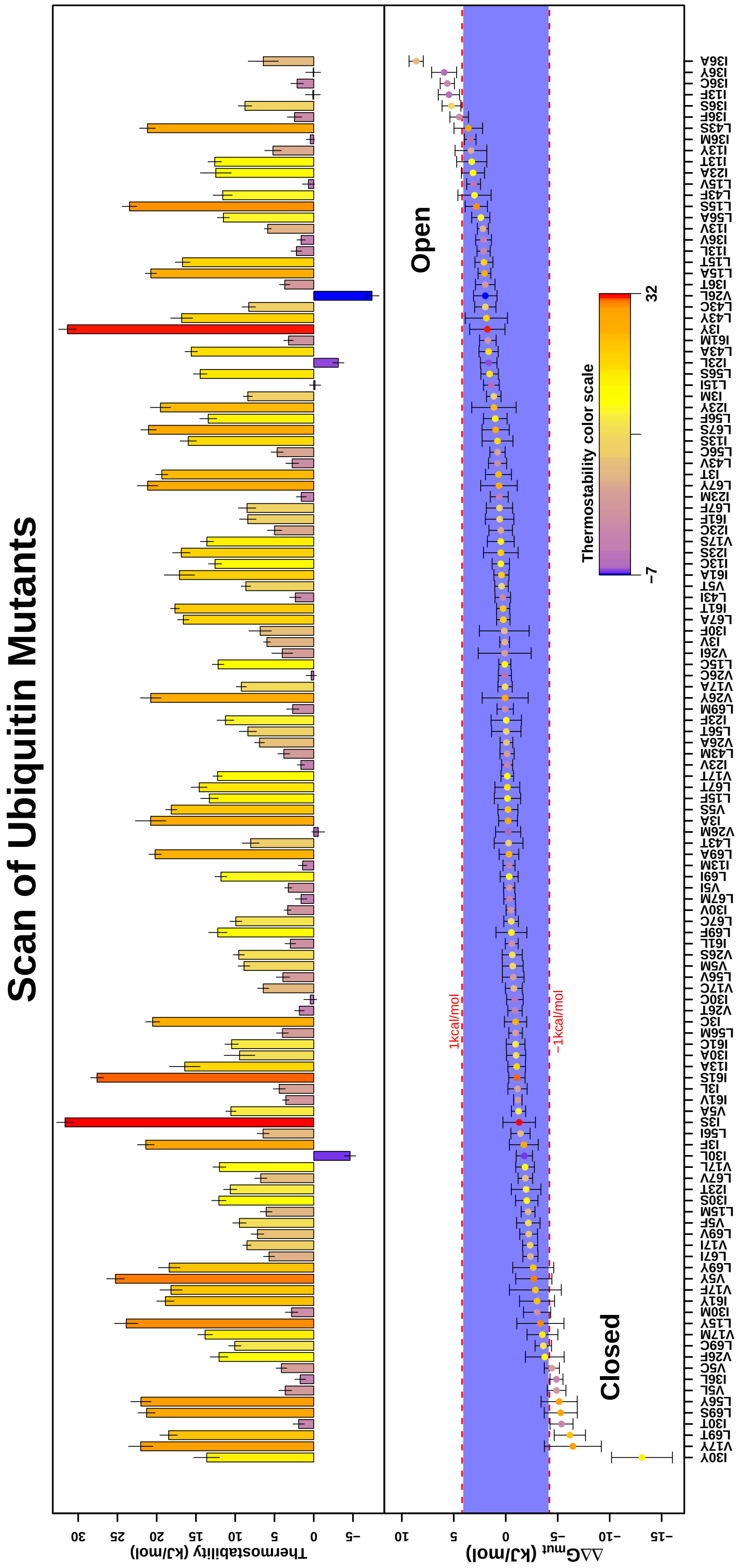

Figure S5: Full results of th FGTI/CGI screening. Both the calculated population shift (bottom) and the change in thermal stability (top) of ubiquitin are shown. The mutations are sorted by the population shift.

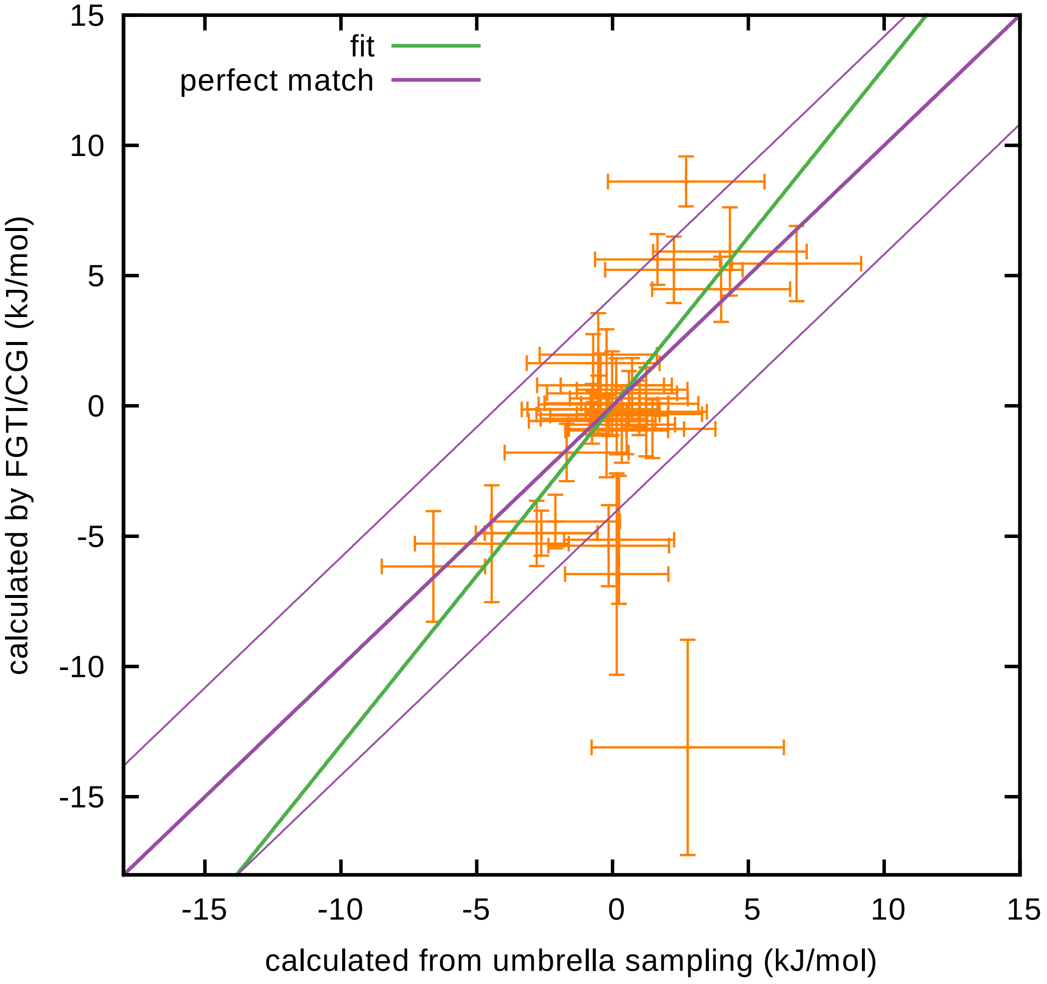

Figure S6: Comparison of open-closed free energy shifts calculated using FGTI/CGI (figure S5) and calculated from potentials of mean force (PMF) (figures S8 and S9). Energy shifts are calculated from PMFs as described in section S1.8. Besides a significant Pearson correlation coefficient $\rho = 0.54$ ($p = 0.0004$), it should be noted that all points where FGTI/CGI and umbrella sampling differ by more than 1 kcal/mol are "false positives", i.e. FGTI/CGI predicts a larger effect than umbrella sampling.

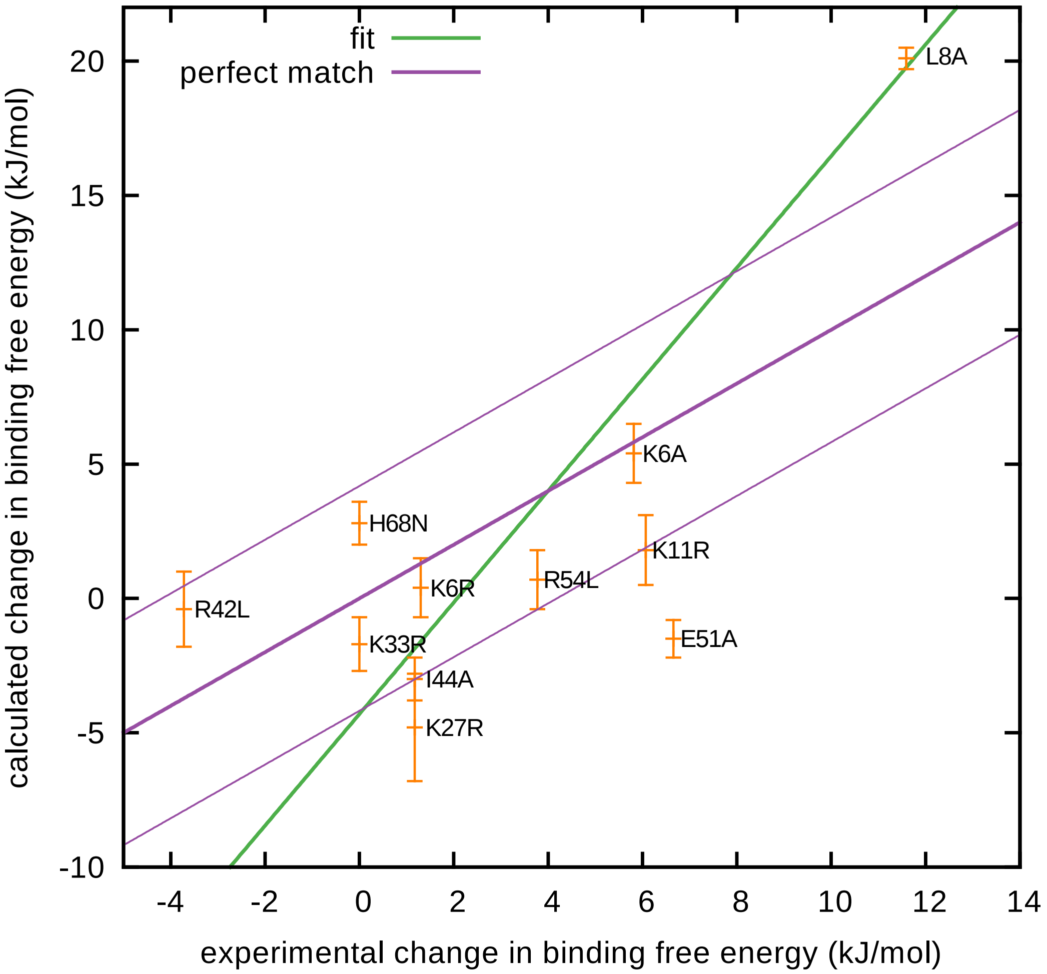

Figure S7: Comparison of experimental and calculated binding free energy changes. The binding free energy differences for ubiquitin binding with UCH-L3 has been measured for different mutations.[23] Here, these values are compared with those calculated using FGTI/CGI. The green line is a fit of the data points. The solid purple line is the perfect match. If the calculations would perfectly reproduce the experimental values, all points would lie on this line. The thin purple lines delimit a deviation of ±1kcal/mol from experimental values. Uncertainties of calculated values have been estimated using bootstrapping. No uncertainties were available for experimental values.

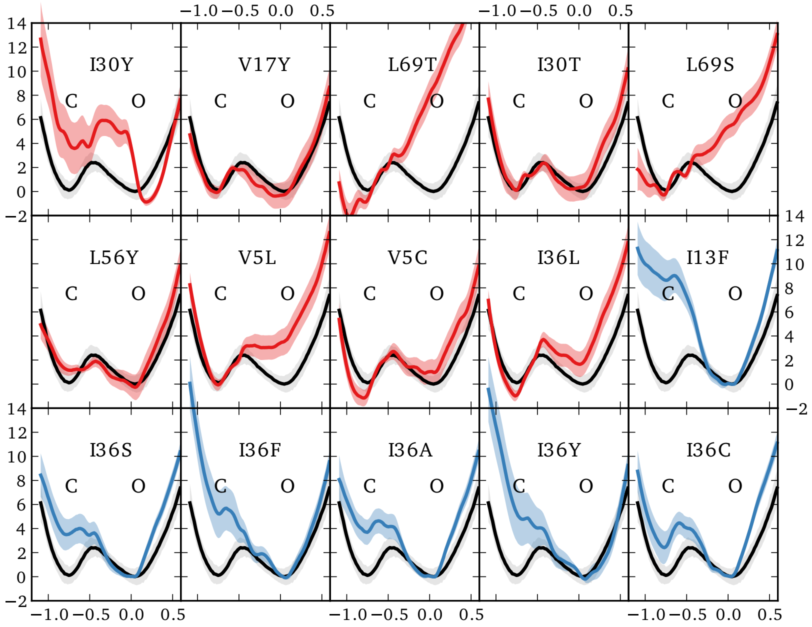

Figure S8: Umbrella sampling simulations for mutations predicted to have a strong effect on the open-closed equilibrium in the initial screening. The free energy profile along the pincer mode is plotted. The plots are ordered according to the predicted population shift (figure S5).

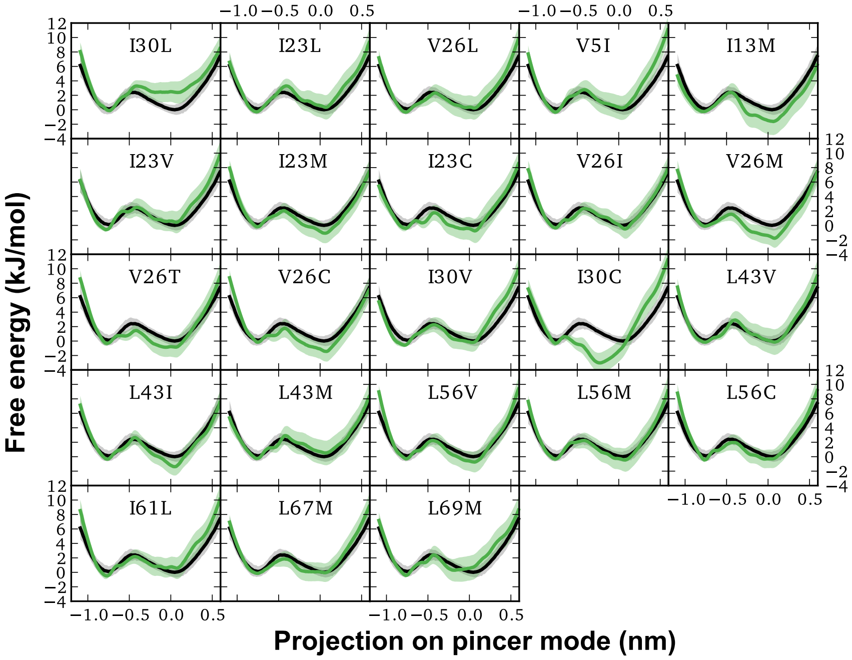

Figure S9: Umbrella sampling simulations for mutations predicted to have limited effect on the open-closed equilibrium (green). The free energy profile along the pincer mode is plotted. For comparison, the wild type free energy profile is plotted for comparison (gray). The plots are ordered according to the predicted population shift (figure S5).

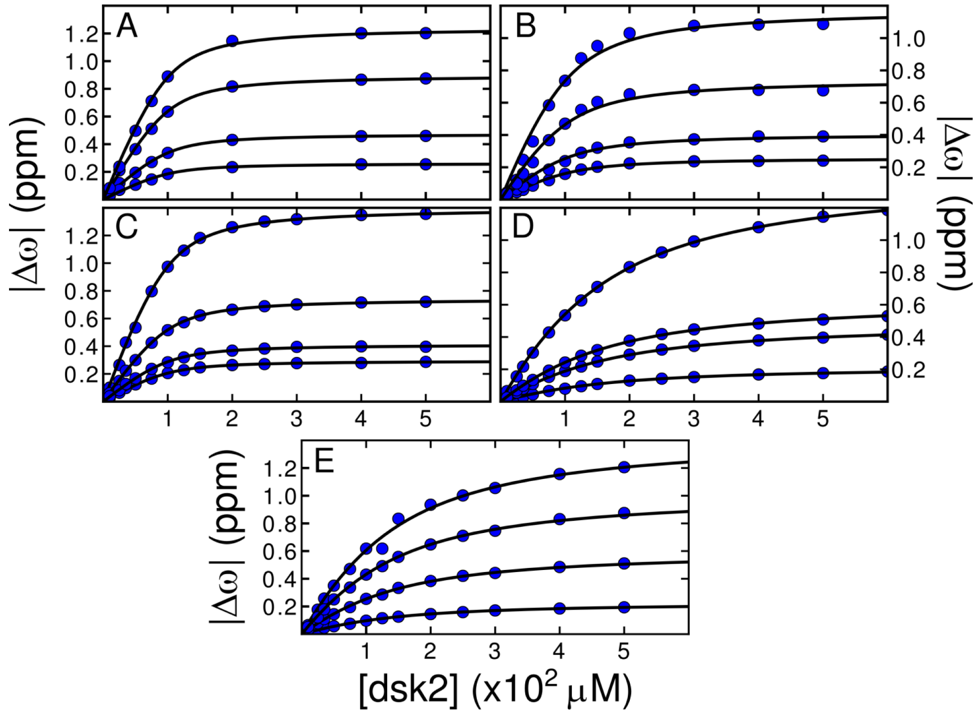

Figure S10: Examples of experimental data depicting the dependence of the ^{15}N chemical shift (ω) as a function of increasing dsk2 concentration for wild type and the examined ubiquitin mutants (A: wild type, B: I13F, C: I36A, D: L69S, E: L69T). The chemical shift difference ($\Delta\omega$) is given by the observed ω at a given concentration of dsk2 subtracted from the ω value for a given ubiquitin mutant resonance with no dsk2 in solution (blue points). Solid black lines designate global fits with a single K_D that assumes a two-state binding model. Each curve represents a different observed resonance from an ubiquitin mutant. Each curve represents a different observed resonance.

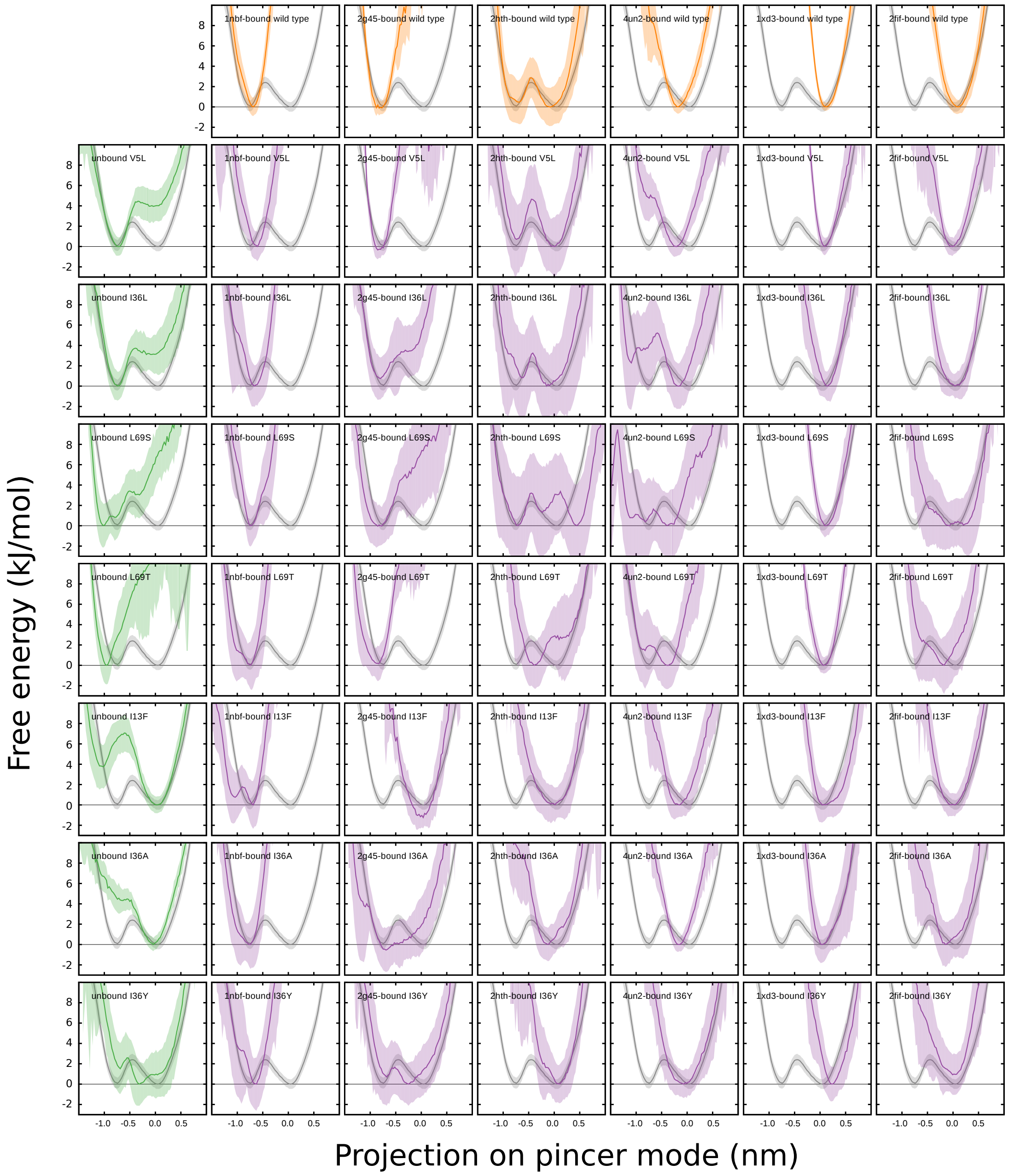

Figure S11: Comparisons of potentials of mean force (PMF) for bound wild type ubiquitin, unbound and bound mutated ubiquitin. PMFs estimated from wild type ubiquitin simulations in the complex are shown in the first row in orange, PMFs calculated from umbrella sampling simulations of unbound mutants are plotted in the first column in green. It can be seen that the PMFs estimated from simulations of mutant ubiquitin in the complex (in violet) are in most cases similar to those of the wild type in the complex, indicating that the binding partner has a stronger influence on the ubiquitin conformation than single point mutations.

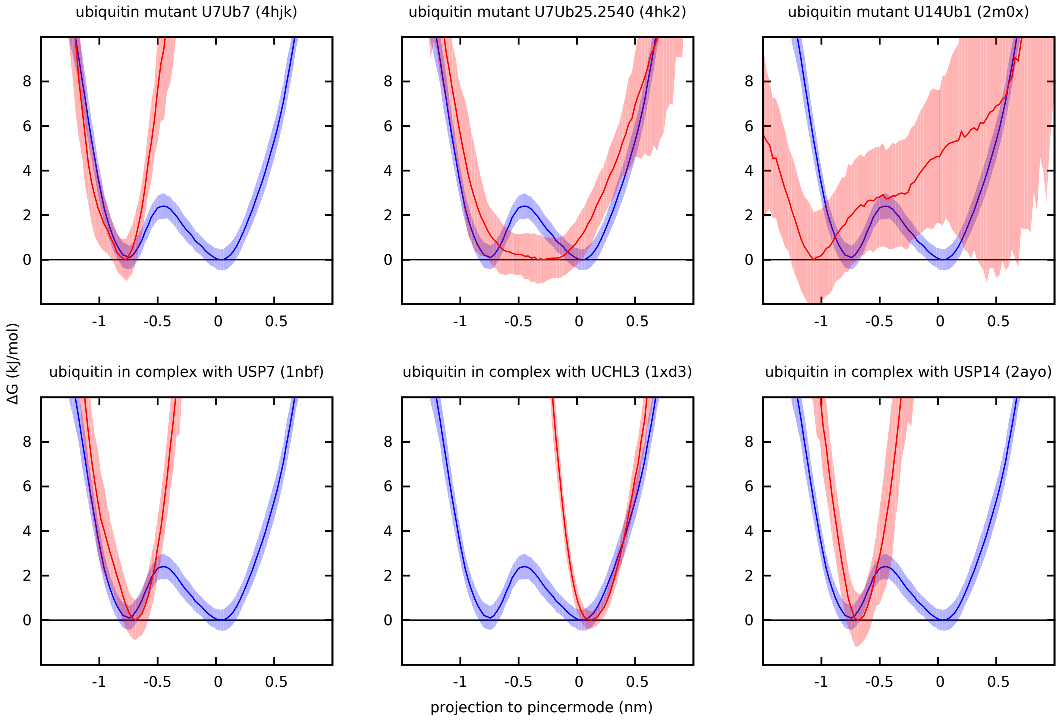

Figure S12: Free energy profiles for selective mutants from the work of Zhang *et al.*[25] and Phillips *et al.*[27] The mutants U7Ub7 and U7Ub25.2540 (upper row, red) have been optimized for increased binding affinity to USP7 (lower left, red) while they do not bind UCHL3 (lower right, red), the mutant and U14Ub1 has been optimized to bind USP14. The profiles have been calculated using the Boltzmann equation from histograms of 10x100 ns (4hjk), 4x10x100 ns (4hk2) or 2x10x100 ns (1nbf,1xd3). For comparison, the free energy profile of unbound ubiquitin has been plotted in each panel (blue line).

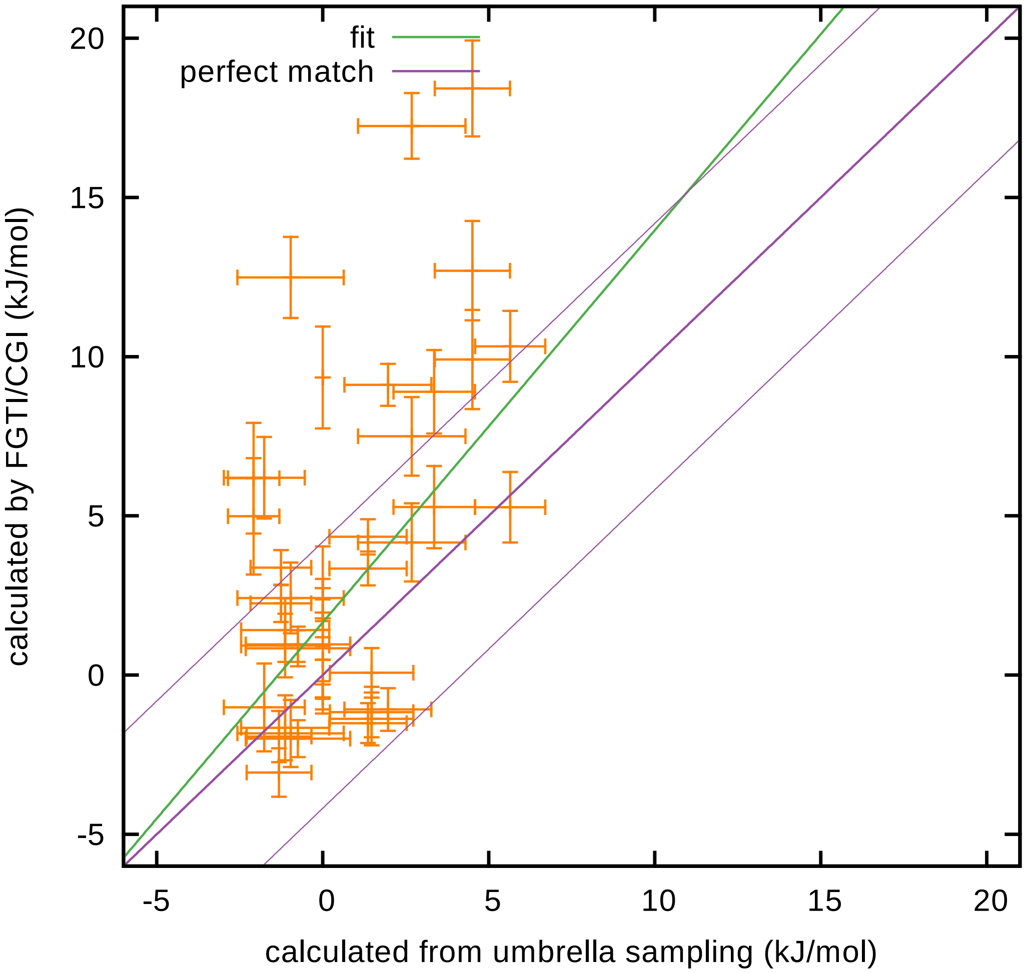

Figure S13: Predicted changes in binding free energy (cf. figure 4 and table S5) compared to the change predicted from the population shift observed in umbrella sampling. Even though the change in binding free energy predicted from the population shift would only be a good prediction of the actual change in binding free energy if this shift was the only effect of the mutation, the correlation of 0.60 is significant (p = 0.00006).

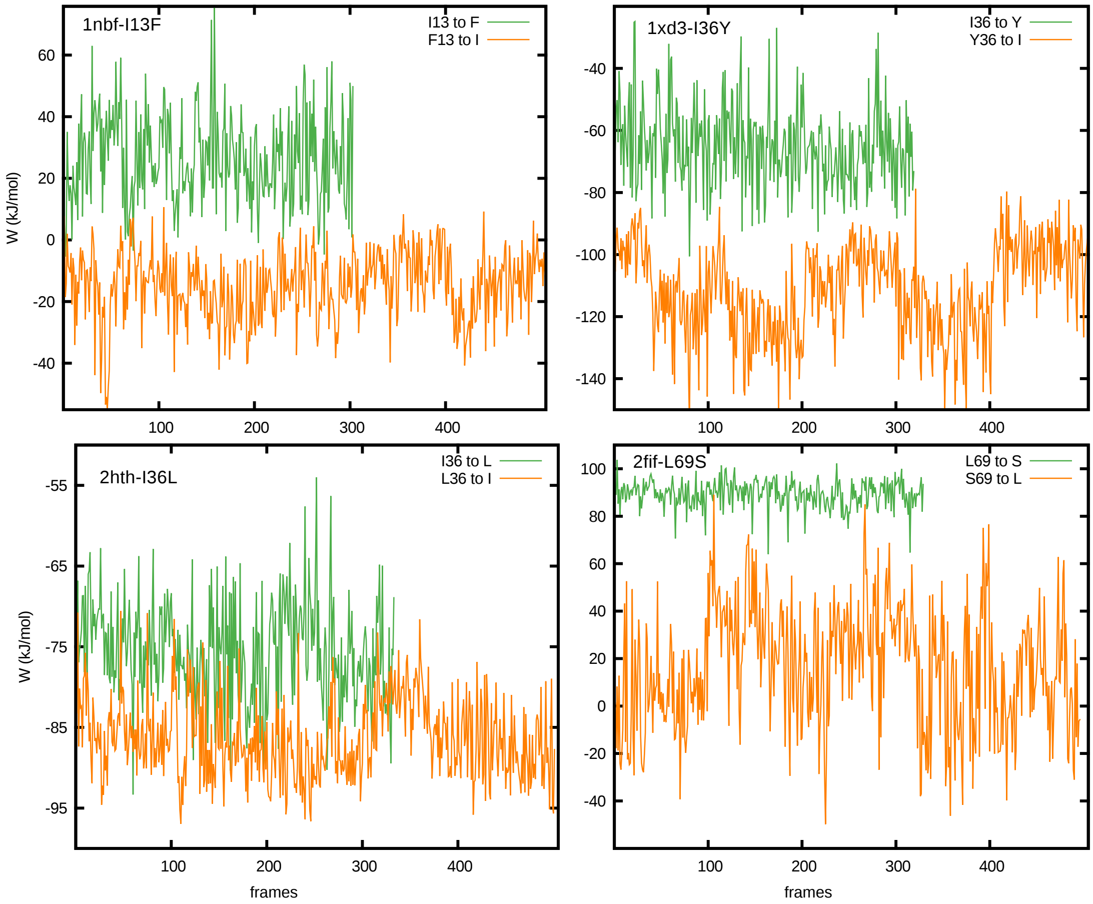

Figure S14: Transition energies underlying CGI calculations for four complex FGTI/CGI calculations. Following the Crooks protocol, snapshots for fast forward and backward transitions were chosen from equilibrium wild-type and mutant trjectories, respectively. The convergence of FGTI/CGI is largely determined by the sampling in these equilibrium runs. If these equilibrium runs suffer from a lack of convergence, this usually translates in patterns/trends in the non-equilibrium work value associated with the snapshots derived from these equilibrium runs. As can be seen in these four exemplary caes, this is not the case in the FGTI/CGI calculations used here.

Table S1: Mutated ubiquitin core residues

Residue number	Residue type	Abbreviation
3	Isoleucine	I3
5	Valine	V5
13	Isoleucine	I13
15	Leucine	L15
17	Valine	V17
23	Isoleucine	I23
26	Valine	V26
30	Isoleucine	I30
36	Isoleucine	I36
43	Leucine	L43
56	Leucine	L56
61	Isoleucine	I61
67	Leucine	L67
69	Leucine	L69

Table S2: Closed cycles on unbound constrained ubiquitin with restrictions to the open/closed substate. All energies are given in kJ/mol.

Wild type residue	mutations	$\Delta\Delta G_{open}$	$\Delta\Delta G_{closed}$
V5	V→L→I→V	-0.1 ± 1.0	1.6 ± 0.8
I13	I→Y→F→I	-1.3 ± 1.2	-3.9 ± 1.2
A15	L→A→M→L	0.9 ± 0.8	-0.7 ± 0.8

Table S3: Closed cycles on ubiquitin complexes. For each cycle, the free energy change for three mutations is calculated. The sum of the three values ($\Delta\Delta G = \Delta G_1 + \Delta G_2 + \Delta G_3$) should be close to zero. All energies are given in kJ/mol.

Complex	residue	Mutations	ΔG_1	ΔG_2	ΔG_3	$\Delta\Delta G$
1xd3	6	K→A→R→K	79.9 ± 0.5	-765.6 ± 1.0	683.6 ± 0.7	-2.1 ± 2.2
1xd3	30	I→T→Y→I	-123.3 ± 0.3	24.6 ± 0.9	98.5 ± 0.8	-0.2 ± 2.1
1nbf	69	L→S→T→L	71.8 ± 0.6	-94.6 ± 0.4	26.8 ± 0.8	4.1 ± 1.8
1nbf	36	I→A→Y→I	18.2 ± 0.3	-108.0 ± 0.7	94.8 ± 0.7	4.9 ± 1.6
1nbf	36	I→A→L→I	18.2 ± 0.3	-92.0 ± 0.3	77.9 ± 0.3	4.1 ± 0.9
1nbf	36	I→L→Y→I	-77.9 ± 0.3	-14.0 ± 0.7	94.8 ± 0.7	2.8 ± 1.6
2g45	69	L→S→T→L	67.7 ± 0.7	-93.8 ± 0.4	24.8 ± 0.6	-1.3 ± 1.7
1xd3	69	L→S→T→L	82.9 ± 0.4	-93.9 ± 0.3	13.4 ± 0.4	2.4 ± 1.1
2fif	69	L→S→T→L	73.1 ± 0.6	-90.7 ± 0.3	19.1 ± 0.5	1.6 ± 1.4

Table S4: Ubiquitin mutants showing a significant difference between the open and closed substates. The free energy difference $\Delta G_{closed} - \Delta G_{open}$ between the mutation restricted to the open and the closed conformation indicates which of the two substates is predicted to be more stable. If the difference is negative, the mutants are predicted to prefer the closed substate, if it is positive, the mutants are predicted to prefer the open substate. For the 15 mutants in this list, umbrella sampling simulations were performed to confirm the predicted preference for one of the substates - the free energy difference between the substates has been calculated as described in section S1.8. For the mutants predicted to prefer the closed substate from screening, a second screening run was perfomed using updated starting structures (section S3). All energies are reported in kJ/mol.

Mutations	$\Delta G_{closed} - \Delta G_{open}$ CGI screening	$\Delta G_{closed} - \Delta G_{open}$ umbrella profile	$\Delta G_{closed} - \Delta G_{open}$ updated CGI screening
I30Y	-13.1 ± 4.1	2.8 ± 3.5	1.8 ± 2.8
V17Y	-6.5 ± 3.9	0.2 ± 1.9	-4.3 ± 2.4
L69T	-6.2 ± 2.1	-6.6 ± 1.9	-8.0 ± 1.5
I30T	-5.4 ± 1.6	-0.1 ± 2.2	0.18 ± 0.9
L69S	-5.3 ± 2.2	-4.4 ± 2.8	-3.1 ± 1.4
L56Y	-5.1 ± 2.5	0.2 ± 2.0	-3.4 ± 1.9
V5L	-4.9 ± 1.3	-2.8 ± 2.2	-2.6 ± 0.8
I36L	-4.9 ± 0.9	-2.6 ± 2.0	-0.35 ± 0.6
V5C	-4.4 ± 1.0	-2.1 ± 2.3	-2.1 ± 0.7
I36F	4.5 ± 1.3	4.0 ± 2.5	
I36S	5.2 ± 1.3	2.2 ± 2.5	
I13F	5.5 ± 1.4	6.8 ± 2.4	
I36C	5.6 ± 1.0	1.7 ± 2.3	
I36Y	5.9 ± 1.7	4.3 ± 2.8	
I36A	8.6 ± 1.0	2.7 ± 2.9	

Table S5: Binding free energy changes due to ubiquitin mutation.

		closed complexes		open complexes			neutral complex
		1nbf	2g45	1xd3	4un2	2fif	2hth
quasi neutral mutants	V5L	-1.9 ± 0.8	-3.1 ± 0.8	-1.4 ± 0.8	0.1 ± 0.8	-1.1 ± 0.8	-0.3 ± 0.8
	I36L	2.2 ± 0.6	3.4 ± 0.5	4.3 ± 0.6	-1.5 ± 0.6	3.3 ± 0.5	-0.7 ± 0.5
closed mutants	L69S	6.2 ± 1.3	-1.0 ± 1.4	17.2 ± 1.0	4.2 ± 1.2	7.5 ± 1.2	2.7 ± 1.3
	L69T	5.0 ± 1.8	6.2 ± 1.7	18.4 ± 1.5	9.9 ± 1.6	12.7 ± 1.2	9.3 ± 1.6
open mutants	I13F	10.3 ± 1.1	5.2 ± 1.1	1.4 ± 1.0	-1.7 ± 1.0	0.9 ± 1.0	2.0 ± 1.1
	I36A	-1.1 ± 0.7	9.1 ± 0.7	1.0 ± 0.6	0.8 ± 0.6	-2.0 ± 0.6	1.8 ± 0.6
	I36Y	5.3 ± 1.3	8.9 ± 1.3	12.5 ± 1.3	2.4 ± 1.1	-1.8 ± 1.1	0.5 ± 1.2

Table S6: Correlation of sidechain motion with pincer mode for the considered core residues. As described in section S1.7, the Pearson correlation coefficient between the pincer mode and a predictive FMA-model based on side chain coordinates of single residues was calculated. A high correlation coefficient indicates relevance of the residue for pincer mode motion. (The mutants have been sorted by the corellation coefficient in descending order)

Abbreviation	correlation coefficient
L69	0.35
I13	0.16
V5	0.15
I36	0.15
L15	0.10
I30	0.09
I3	0.04
L56	0.00
L67	-0.01
V17	-0.03
V26	-0.04
I61	-0.05
L43	-0.06
I23	-0.08

References

(1) Hess, B.; Kutzner, C.; van der Spoel, D.; Lindahl, E. *Journal of Chemical Theory and Computation* **2008**, *4*, 435–447.

(2) Cornell, W. D.; Cieplak, P.; Bayly, C. I.; Gould, I. R.; Merz, K. M.; Ferguson, D. M.; Spellmeyer, D. C.; Fox, T.; Caldwell, J. W.; Kollman, P. A. *Journal of the American Chemical Society* **1995**, *117*, 5179–5197.

(3) Berendsen, H. J. C.; Grigera, J. R.; Straatsma, T. P. *The Journal of Physical Chemistry* **1987**, *91*, 6269–6271.

(4) Joung, I.; III, T. C. *J. Phys. Chem. B* **2008**, *30*, 9020–9041.

(5) Bussi, G.; Donadio, D.; Parrinello, M. *The Journal of Chemical Physics* **2007**, *126*, 014101.

(6) Berendsen, H. J. C.; Postma, J. P. M.; van Gunsteren, W. F.; DiNola, A.; Haak, J. R. *The Journal of Chemical Physics* **1984**, *81*, 3684.

(7) Essmann, U.; Perera, L.; Berkowitz, M. L.; Darden, T.; Lee, H.; Pedersen, L. G. *The Journal of Chemical Physics* **1995**, *103*, 8577.

(8) Miyamoto, S.; Kollman, P. A. *Journal of Computational Chemistry* **1992**, *13*, 952–962.

(9) Hess, B.; Bekker, H.; Berendsen, H. J. C.; Fraaije, J. G. E. M. *Journal of Computational Chemistry* **1997**, *18*, 1463–1472.

(10) Hess, B. *J. Chem. Theory Comput.* **2008**, *4*, 116–122.

(11) Goette, M.; Grubmüller, H. *Journal of Computational Chemistry* **2009**, *30*, 447–456.

(12) Amadei, A.; Linssen, A. B. M.; de Groot, B. L.; van Aalten, D. M. F.; Berendsen, H. J. C. *Journal of Biomolecular Structure and Dynamics* **1996**, *13*, 615–625.

(13) de Groot, B. L.; Amadei, A.; Scheek, R. M.; a J van Nuland, N.; Berendsen, H. J. C. *Proteins: Structure, Function, and Bioinformatics* **1996**, *26*, 314–322.

(14) de Groot, B. L.; Amadei, A.; van Aalten, D. M. F.; Berendsen, H. J. C. *Journal of Biomolecular Structure and Dynamics* **1996**, *13*, 741–751.

(15) Vriend, G. *Journal of Molecular Graphics* **1990**, *8*, 52–56.

(16) Seeliger, D.; de Groot, B. L. *Biophys. J.* **2010**, *98*, 2309–2316.

(17) Kumar, S.; Rosenberg, J. M.; Bouzida, D.; Swendsen, R. H.; Kollman, P. A. *Journal of Computational Chemistry* **1992**, *13*, 1011–1021.

(18) Hub, J. S.; de Groot, B. L.; van der Spoel, D. *Journal of Chemical Theory and Computation* **2010**, *6*, 3713–3720.

(19) Lazar, G. A.; Desjarlais, J. R.; Handel, T. M. *Protein Science* **1997**, *6*, 1167–1178.

(20) Markin, C. J.; Spyracopoulos, L. *J. Biomol. NMR* **2012**, *54*, 355–376.

(21) Hub, J. S.; de Groot, B. L. *PLoS Computational Biology* **2009**, *5*, e1000480.

(22) Krivobokova, T.; Briones, R.; Hub, J. S.; Munk, A.; de Groot, B. L. *Biophysical Journal* **2012**, *103*, 786–796.

(23) Wilkinson, K. D.; Laleli-Sahin, E.; Urbauer, J.; Larsen, C. N.; Shih, G. H.; Haas, A. L.; Walsh, S. T. R.; Wand, A. J. *Journal of Molecular Biology* **1999**, *291*, 1067–1077.

(24) Haririnia, A.; Verma, R.; Purohit, N.; Twarog, M. Z.; Deshaies, R. J.; Bolon, D.; Fushman, D. *J. Mol. Biol.* **2008**, *375*, 979–996.

(25) Zhang, Y.; Zhou, L.; Rouge, L.; Phillips, A. H.; Lam, C.; Liu, P.; Sandoval, W.; Helgason, E.; Murray, J. M.; Wertz, I. E.; Corn, J. E. *Nat. Chem. Biol.* **2013**, *9*, 51–58.

(26) Peters, J. H.; de Groot, B. L. *PLoS Comput. Biol.* **2012**, *8*, e1002704.

(27) Phillips, A. H.; Zhang, Y.; Cunningham, C. N.; Zhou, L.; Forrest, W. F.; Liu, P. S.; Steffek, M.; Lee, J.; Tam, C.; Helgason, E.; Murray, J. M.; Kirkpatrick, D. S.; Fairbrother, W. J.; Corn, J. E. *Proc. Natl. Acad. Sci. USA* **2013**, *110*, 11379–11384.
